# Camel Proteins and Enzymes: A Growing Resource for Functional Evolution and Environmental Adaptation

**DOI:** 10.3389/fvets.2022.911511

**Published:** 2022-07-12

**Authors:** Mahmoud Kandeel, Abdulla Al-Taher, Katharigatta N. Venugopala, Mohamed Marzok, Mohamed Morsy, Sreeharsha Nagaraja

**Affiliations:** ^1^Department of Biomedical Sciences, College of Veterinary Medicine, King Faisal University, Al-Ahsa, Saudi Arabia; ^2^Department of Pharmacology, Faculty of Veterinary Medicine, Kafr El Sheikh University, Kafr El Sheikh, Egypt; ^3^Department of Pharmaceutical Sciences, College of Clinical Pharmacy, King Faisal University, Al-Ahsa, Saudi Arabia; ^4^Department of Biotechnology and Food Science, Faculty of Applied Sciences, Durban University of Technology, Durban, South Africa; ^5^Department of Clinical Sciences, College of Veterinary Medicine, King Faisal University, Al-Ahsa, Saudi Arabia; ^6^Department of Surgery, Faculty of Veterinary Medicine, Kafr El Sheikh University, Kafr El Sheikh, Egypt; ^7^Department of Pharmacology, Faculty of Medicine, Minia University, Minya, Egypt; ^8^Department of Pharmaceutics, Vidya Siri College of Pharmacy, Bengaluru, India

**Keywords:** camel, metabolism, adaptation, enzymes, proteins

## Abstract

In less agroecological parts of the Asian, Arabian, and African deserts, *Camelus dromedarius* play an important role in human survival. For many years, camels have been employed as a source of food, a tool of transportation, and a means of defense. They are becoming increasingly important as viable livestock animals in many desert climates. With the help of camel genetics, genomics and proteomics known so far, this review article will summarize camel enzymes and proteins, which allow them to thrive under varied harsh environmental situations. An in-depth study of the dromedary genome revealed the existence of protein-coding and fast-developing genes that govern a variety of metabolic responses including lipid and protein metabolism, glucoamylase, flavin-containing monooxygenase and guanidinoacetate methyltransferase are other metabolic enzymes found in the small intestine, liver, pancreas, and spleen. In addition, we will discuss the handling of common medications by camel liver cytochrome p 450, which are different from human enzymes. Moreover, camels developed several paths to get optimum levels of trace elements like copper, zinc, selenium, etc., which have key importance in their body for normal regulation of metabolic events. Insulin tolerance, carbohydrate and energy metabolism, xenobiotics metabolizing enzymes, vimentin functions, behavior during the rutting season, resistance to starvation and changes in blood composition and resistance to water loss were among the attractive aspects of camel enzymes and proteins peculiarities in the camels. Resolving the enigma of the method of adaptation and the molecular processes linked with camel life is still a developing repository full of mysteries that need additional exploration.

## Introduction

Humans are greatly reliant on camels, who provide support in a variety of ways, particularly in their arid habitat. Camel has shown to be a vital economic component in recent years, generating significant earnings for the country's economy (1). Camels are considered one of the most powerful land mammals on the planet due to their amazing ability to flourish in arid settings. Camels, which are members of the Camelidae family, were originally discovered in northern America about 35 million years ago during the Eocene period (2, 3).

There are a variety of camel breeds. Even though their shape is almost identical, the differences between them may be seen most clearly in their sizes, colors, and conformation (3).

The Camelidae family includes two primary types of camels, small and large camels, which are further subdivided into genera such as Camelus, Lama, and Vicugna. Among large camels, two domestic species are more commonly known as *Camelus bactrianus* and *Camelus dromedarius* having two humps and a single hump, respectively. Camelus dromedarius is also known as the Arabian camel, and this animal species is most usually seen in northern Africa, where the habitats are dry and have extreme weather conditions (4).

Because of their capacity to maneuver through the desert with large-weight loads, they are known as “ships of the desert.” Camels are highly vital animals in many countries for meat because they contain high levels of protein and low lipid content, secondly for milk because their milk contains specific substances that are effective against a wide range of diseases, and thirdly for their skin, which is used in many leather industries as a source of warm and shiny leather. Camel milk has particular immunoprotective substances that can activate immunological and molecular processes against certain biological illnesses (5).

## Camel's Unique Genomics

Camel farming is important for a variety of reasons, including economic, cultural, and biological considerations. Unfortunately, there hasn't been any in-depth research on their genomes. Camels have a lengthy history of evolutionary advantages that have yet to be completely explored, despite their popularity. In the past, it has been demonstrated that domestic and livestock animals exhibit diverse features as a result of genetic differences (6, 7). In 2012, the first genome sequence providing information on domestic and wild Bactrian camels was released. In the same year, the Bactrian camel's entire genome, with 20,821 genes and a total size of 2.38GB, was published. Other than general and genetic investigations, the remainder of the studies has explained the unexpected living habits of camels (6–8). Many genes which are responsible for species differentiation and unusual adaptions in camels evolve rapidly (9, 10). The most essential coding genes for proteins in different species, as well as their rapid divergence, are often estimated using a method published in prior studies (10).

Camels have a very unique variety of genome that contains 20,000 genes approximately, on a total sized 2.38 GB genome. The repeated sequence is 28.2% in the dromedary camel genome, which is 14–18% lower than cattle and human genomes. Four Cetartiodactyla species (Bactrian camel, dromedary, alpaca, and cattle) shared 12,539 homologous gene families. The Bactrian camel, dromedary, and alpaca each had unique 156, 153, and 296 gene families, respectively (8). In addition to helping them adapt to the severe climatic conditions on land, this informal genetic makeup supports the camel genome in repairing a wide range of biological ailments.

It has also been discovered that the Camelus dromedarius genome contains a number of fast-developing genes that enable camels to withstand harsh desert conditions (Table 1). Camelus dromedarius transcriptomics and genomes have also revealed the distinct adaptations of these species separate from the physiological changes (10). Moreover, these protein-coding genes were involved in various types of metabolic processes like lipid and carbohydrate metabolism, adipocyte signaling pathways, and insulin signaling pathways. Mitochondrial enzymes of camels have a high evolution rate hence they adapted to live in different environments (36, 37).

**Table 1:** The unique aspects of camel genomics, proteomics and adaptation mechanisms.

**Aspect**	**Description**	**Reference**
Genome repeated sequence	The repeated sequence is about 14–18% lower than cattle and human genomes.	(8)
New gene families	The Bactrian camel, dromedary, and alpaca each had unique 156, 153, and 296 gene families	(8)
Gene evolution	About 2,730 faster-evolving genes in lipid and carbohydrate metabolism, adipocyte signaling pathways, water balance, metabolism and insulin signaling pathways.	(10)
Immunology	Camel heavy chain antibodies and camel nanobodies for wide application in diagnostics and therapeutics	(12, 13)
Water loss	*Camelus dromedarius* may lose up to 25% of its body weight in water under acute dehydration without risking its health.	(6, 7)
Erythrocytes	Camel erythrocytes may grow up to 240 percent of their original size without bursting. As a result, camels are very resistant to osmotic hemolysis.	(14)
Erythrocytes	Altered distribution of membrane phospholipids	(15)
Kidneys	It has a high capacity for water reabsorption and excretes high concentration urine.	(16)
Small intestine	Less loss of water in excreta by higher water absorption capacity.	(16)
Body temperature	The normal range is 34 and 41 degrees Celsius according to the surrounding circumstances	(17)
Sweating	Camels start to sweat only when their body temperature exceeds 42 degrees Celsius	(18)
Carbohydrase enzymes	High-efficiency enzymes with higher energy assimilation and storage capability	(19)
CYP2J	Bactrian camels, cows, horses, and humans have 11, 4, 1, and 1 copies, respectively. Larger number in camel.	(10)
CYP2E	Bactrian camels, cows, horses, and humans have 2, 1, 1, and 1 copies, respectively. Larger number in camel.	(10)
CYP4A	Bactrian camels, cows, horses, and humans have 2, 3, 3, and 2 copies, respectively. Fewer number in camel.	(10)
CYP4F	Bactrian camels, cows, horses, and humans have 2, 7, 7, and 6 copies, respectively. Fewer number in camel.	(10)
Higher CYP2J CYP2E copies and lower CYP4A and CYP4F copies	Maintains 19(S)-HETE, which is a powerful vasodilator of renal preglomerular arteries that promotes water absorption	(10)
CYP2J2	Downregulated during high salt diet in rats. The multiple copies in camel might help in maintaining blood osmolarity and vasodilatation of renal blood vessels	(20)
α-actin	Overexpression in camel myocytes, an adaptive trait for supporting hemoconcentration–hemodilution phases associated with alternating drought–rehydration periods.	(21)
β-crystallin	Overexpressed in camel heart, improves protein folding and cellular regeneration in the dromedary heart.	(21)
H^+^-ATPase	Overexpressed in camel brain, provide an alternative quick source of energy supply	(21)
Guanidinoacetate methyltransferases	Help in maintaining a constant nitrogen level by urea-nitrogen recycling while withstanding starvation and antioxidant	(22)
testosterone and 5α-dihydrotestosterone	Elevated in serum during the rutting season.	(23)
Leydig cell number per testes	Increases during rutting season for the production of a larger amount of androgens.	(24)
5α-DHT	Overexpressed in rutting season and more reactive than testosterone	(25)
Vimentin	The up-regulation of vimentin in adipocytes, boosting lipoprotein translocation, blood glucose trapping, and confronting external physical extra-stress, create a dynamic character for camel hump adipose tissue.	(21)
Vimentin	Response to stimulation of adrenergic receptors and lipolysis.	(26)
Flavin-containing monooxygenase (FMOs)	Similar to humans, camel FMO-catalyzed metabolism was independent of cytochrome CYPs activity.	(27)
Minerals	Higher storage capacity of copper	(28)
	During the scarcity period more absorption of zinc and copper	(29)
	Maintaining normal enzymatic activity throughout the malnutrition phase, as well as tolerance for excess electrolytes and minerals such as sodium, calcium and phosphorous.	(30)
Selenium	Camel RBCs to store selenium during deficiency periods	(31)
Carbohydrase	Camel pancreas and intestine express efficient carbohydrate metabolizing enzymes	(32)
Natural insulin resistance	Higher CYP2E activity is associated with type II diabetes Miletus.	(33)
	Upregulation of 21 genes in insulin and type II diabetes signaling pathways	(10)
	Elevated glucagon level in camels	(34)
	Insulin receptor structural differences in camels.	(35)

The genetic diversity and genetic selection of camels have been recently studied (38, 39). The complete genome sequence data of six Arabian Peninsula camels and the genotyping-by-sequencing data of 44 Sudanese camels (29 packing and 15 racing) were studied to assess their genome diversities, relationships, and possible signals of positive selection (11). The Sudanese and Arabian Peninsula camels are clearly separated geographically, yet there is no population-specific genetic differentiation within populations. In order to discover and describe genes and variants connected to this beneficial phenotypic characteristic, additional study of the dromedary camel genome is encouraged in light of these discoveries. As a result, breeding programs aimed at improving the unique domesticated species' output and performance could benefit from the findings. In another study, genetic diversity among camel types was low, with the highest measure of genetic diversity found in Targui and the lowest in Awarik; camel types from Asia (especially the Arabian Peninsula) exhibited higher genetic diversity than their African counterparts (40).

## Phenotypical and Environmental Adaptation in Camels

To live in desert harsh conditions camels have modified their living lifestyles because of special genotypes and phenotypes. In comparison to some other mammals, they have developed their senses more efficiently including visionary organs, sniffing organs, body water balance and metabolism, and mechanisms of heat control are a few most highlighted ones (16). The fats in the camel hump are a rich source of not only fats but also proteins which develop a well-organized cytoskeleton. These highly organized cytoskeletons help camels in movement in desert sands. Camels are known to possess the highest blood sugar levels among ruminants (21).

**Figure 1:**
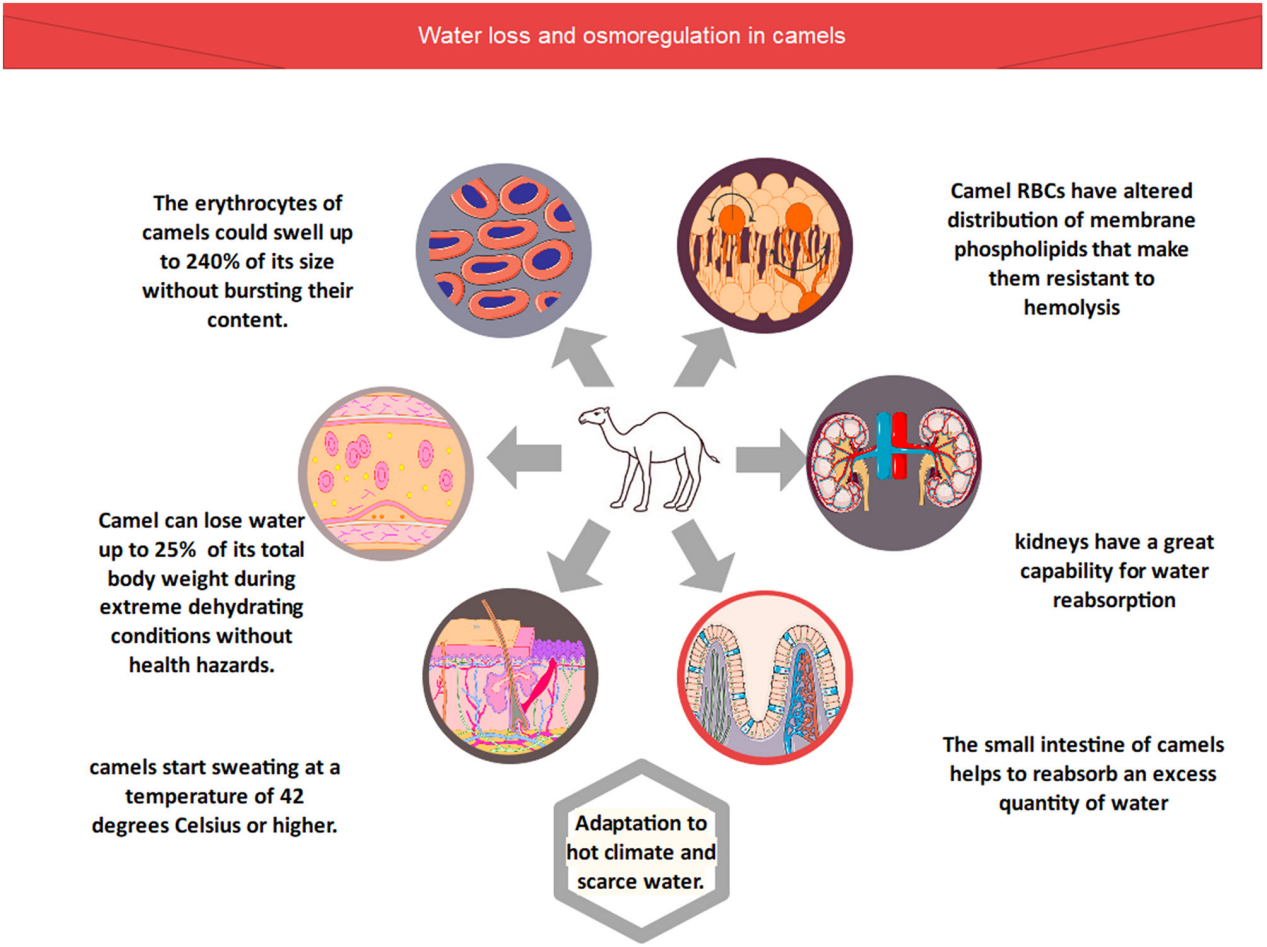
Water loss and osmoregulation in camels.

In the body of the camel, different types of processes, including blood osmolarity and tissue osmolarity (Figure 1), regulate the optimum concentration of salts and water (6). *Camelus dromedarius* could lose 25% water of its total body weight during extreme dehydrating conditions without health hazards. In comparison, other animal species could not survive in these conditions due to the failure of their circulatory system when water excretion is more than 12% of their total body weight (6, 7). However, camels possess a unique characteristic of losing more than 25% water of their total body weight due to these physiological adaptations. Therefore, they can easily survive in dry conditions (6, 7). Camels' erythrocytes, on the other hand, can expand up to 240 percent of their original size without bursting. As a result, these organisms are extremely resistant to hemolysis due to osmotic pressure (14). Furthermore, the composition of red blood cells revealed that they contained altered distribution of membrane phospholipids that make them resistant to hemolysis (15). Furthermore, the kidneys of *Camelus dromedarius* have also physiologically adapted for extra conservation of water by upregulating the process of osmolarity of urine. Furthermore, their kidneys have a great capability for water reabsorption at the same time, and it excretes high-concentration urine. Furthermore, as compared to other animal species, the small intestine of camels helps to reabsorb an excess quantity of water. As a result, camels lose less water in their feces, which is why their feces are so dry (16).

The body temperature of the camel ranges between 34 and 41 degrees Celsius, as recorded using thermographic tracing (17). In general, the most dramatic changes in camel body temperature are seen between the early hours of the morning and the late hours of the afternoon. Camels are able to regulate their body temperature in order to avoid water loss through perspiration in reaction to these temperature variations. Even so, this does not negate the fact that camels perspire at a temperature of 42 degrees Celsius or higher. In hot weather, camels begin to sweat when the temperature reaches this level (17, 18).

The dromedary can live in dry regions, near mountains, and distant locations because of specific qualities and attributes that allow them to thrive in desert lands, mountains, and remote areas where food and water are scarce (41). Different sorts of metabolic processes occur in camels' bodies, which are primarily governed by the correct activities of different types of enzymes (19). Carbohydrase enzymes, including amylase, disaccharidases, lipases, maltase, glucoamylase and hydrolytic enzymes, are present in the small intestine and pancreas of a dromedary, which helps in the digestion of food properly additionally these enzymes help to store the extra amount of energy that will further utilized during extreme conditions. That's why this abstemious ruminant is capable of fortitude during hunger and thirst in inhospitable ecological areas (19).

## Camel Cytochrome P450

Cytochrome P450 enzymes are a type of monooxygenase with a prosthetic group of heme-iron (Type-B). From microbes to humans, these enzymes can be found in all kingdoms. Membrane-bound enzymes are normally located on the endoplasmic reticulum's outer surface in humans, however some of these enzyme families can also be found in mitochondria. Human P450s enzymes perform a range of tasks, the most important of which are *N*-dealkylation, aromatic and aliphatic hydroxylation, and epoxidations (42). There are 57 distinct P450s present in humans (43). About 13 of these 57 P450s are classified as “orphan p450s” since no metabolic activity has been described for them, implying that they do not metabolize any physiological substrates or other xenobiotics (43). All fields of science including biochemistry, biotechnology, enzymology, microbiology, pharmacology, plant sciences, and toxicology have a wide range of interest and applications in P450s. Cytochrome P450 (CYPs) are highly specific and versatile biocatalysts that perform diverse reactions (44). They also show significant stereospecificity and regiospecificity for molecules with different structures and compositions. The key metabolic pathways of P450 aid in phase I metabolism. When a hydroxyl (-OH) group is added to the aliphatic or aromatic rings of xenobiotics, they become more polar hydroxylated molecules. These hydroxylated products are then metabolized by Phase-II enzymes, which glucuronidate the hydroxylated phase-I products, and the body excretes the final metabolites in urine or bile.

**Figure 2:**
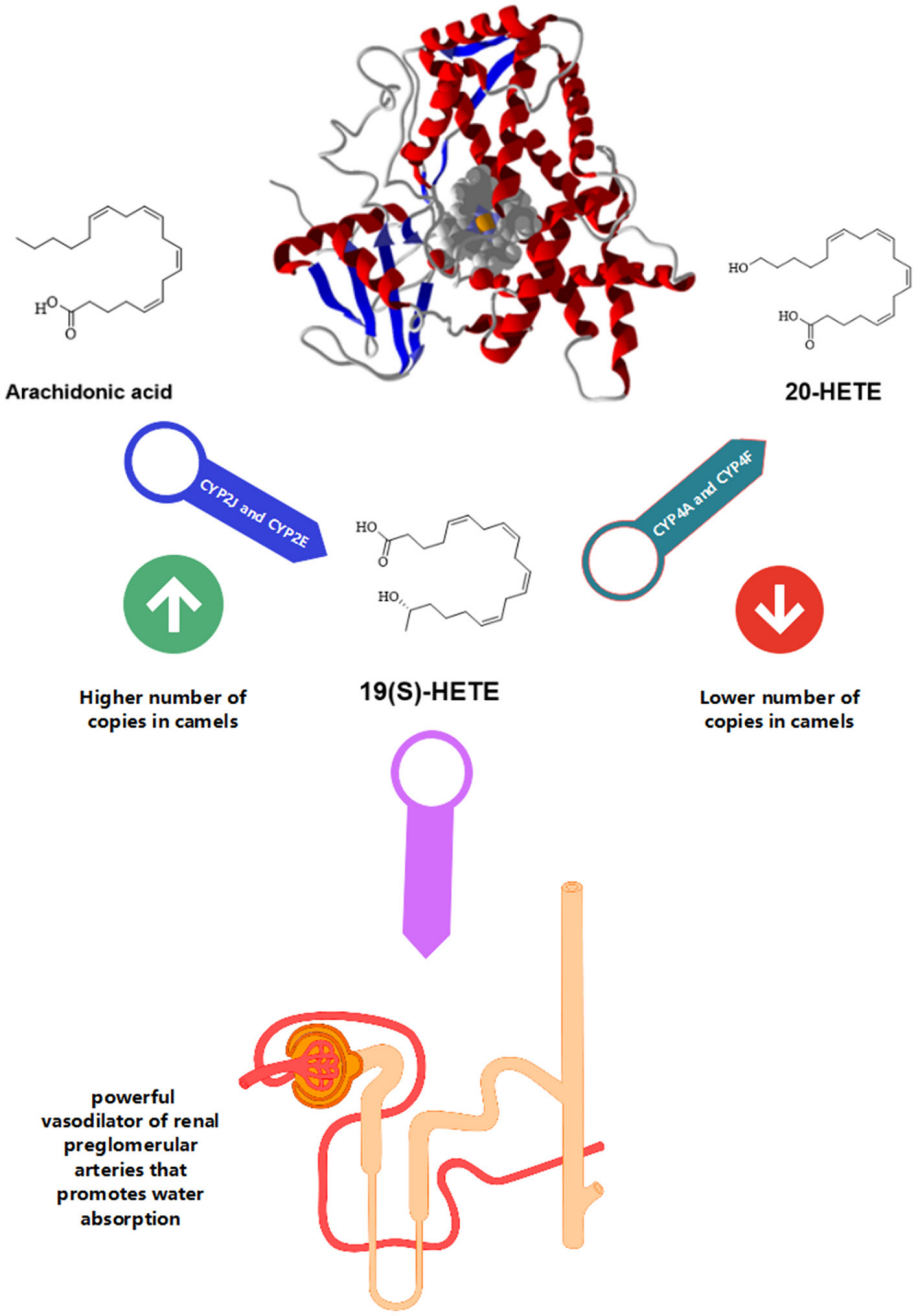
Camel CYP450. The compound 19(S)-HETE is a powerful vasodilator of renal preglomerular arteries that promotes water absorption. There is a higher number of copies of CYP2J and CYP2E and a lower number of copies for CYP4A and CYP4F.

Compared to other animals, the distribution of cytochrome P450 (CYP) genes that regulate the arachidonic acid metabolism was significantly disparate in camels. When compared to closely related animals and humans (Figure 2), Bactrian camels have a larger number of copies of CYP genes such as CYP2J (11 copies) and CYP2E (2 copies) in their genomes. However, there were fewer copies of CYP4F (two copies) and CYP4A (one copy) found in camels (3, 10). Arachidonic acid is converted into [19(S)-HETE] by CYP2E and CYP2J genes, whereas it is converted to 20-HETE by CYP4F and CYP4A. Interestingly, 19(S)-HETE is a powerful vasodilator of renal preglomerular arteries that promotes water absorption that significantly helps these species for desert survival. Extra and deficient copies of CYPs in camels are therefore a crucial aspect of adaptation. Additionally, these species also possess the ability to store high concentrations of salt without developing hypertension due to the presence of multiple copies of CYP2J genes. Therefore, high salt diets influence CYP2J2 activity, and the suppression of this gene could result in high blood pressure (20).

Because there have been few previous investigations, the liver metabolic enzymes of camels are still unclear. A few medications, like monensin and salinomycin, cause rapid intoxication in camels, and the genomic material responsible for this intoxication must be investigated. There are several camel CYPs that evolve at a far slower pace than human CYPs. Camel CYP1A1 is the most prevalent cytochrome P450 among them. Attempts to research the camel genome in-depth and redeem a few CYPs of camels, including cytochrome CYP1A1, 2C, and 3A enzyme (45). Some medicines, such as monensin, alfanaphthoflavone, salinomycin, ritonavir, and felodipine, have been reported to have very poor binding affinities with camel cytochrome P450s (45). Studies using rerank scores revealed that salinomycin and monensin had very weak affinities for camel CYP1A1 and salinomycin has very weak affinities for camel CYP2C. Both drugs exhibit weak interactions with the cytochrome P450s listed above. This means that camels are more vulnerable to the hazardous effects of these drugs in arid and desert conditions (45). Unlikely various drugs cause intoxication in only camels in comparison to other closely related families (46–48). For example, camels are unable to withstand ionophores such as salinomycin and monensin (48, 49). Chicken usually resists up to 50 mg/kg, whereas 0.6 mg/kg dose rate is lethal for camels. Similar studies showed that diminazene aceturate was found to be less toxic in other species compared to camels (50). The drug metabolism nature and camel genetic background associated with it has not been investigated. It's still not known if cytochrome P450 of camel is evaluated or adapted for the desert environment. Monensin was metabolized mainly by the oxidation of CYPs (51, 52). Lower evolution rate and camels CYPs drug-binding capacity compared to the human enzymes could be a reason beyond camel toxicity with these compounds.

## Renal Blood Flow and Renal Vasodilatation

Arachidonic acid is converted to 19(S)-hydroxy-eicosatetraenoic acid [19(S)-HETE] by CYP2J and CYP2E. While CYP4F and CYP4A help in the transformation of arachidonic acid to 20-HETE. Renal preglomerular vessels aid in water reabsorption and are necessary for life in desert environments. 19(S)-HETE is potent vasodilator of this vessel (10). Administration of angiotensin II and high salt diet downregulated CYP2J2 in renal vessels leading to hypertension (20). Therefore, due to numerous copies of the CYP2J gene, the camel can consume a considerable amount of salt without developing hypertension (3).

Elevated serum urea and creatinine levels have been reported during dehydration in camels (53, 54). Camels undergoing dehydration for 20 days showed a significant rise in plasma creatinine, serum sodium, plasma arginine vasopressin (AVP), and urea levels. The antidiuretic hormones system which includes aldosterone and antidiuretic hormone showed little changes (55). These findings support former studies which show that the renin-angiotensin mechanism is vital for water balance maintenance to control dehydration.

## Resistance to Fluctuations in Blood Concentration

The relative overexpression of α-actin in the dromedary heart compared to the rat heart suggests an adaptive trait for supporting hemoconcentration-hemodilution phases associated with alternating drought–rehydration periods. Furthermore, increased β-crystallin expression, a minor heat shock protein, improves protein folding and cellular regeneration in the dromedary heart (21).

## Enzymes in Camels and Resistance to Starvation

Camel can withstand water and feed deprivation, compared with other animal species (56). Camel have evolved rapidly developed genes concerned with stress resistance, comprising DNA damage and repair, apoptosis, protein stabilization, oxidoreductase activity, enriched in cytochrome c oxidase and and monooxygenase activities (8). Urea recycling in camels was consistently high (94-−97%), and nitrogen balance did not change with water deprivation (56). Camels also can maintain a constant nitrogen level by urea-nitrogen recycling while withstanding starvation (3). Camel liver has several enzymes, which have regulatory effects on high-energy phosphate molecules. Among these enzymes, guanidinoacetate methyltransferases are the most common, which perform these functions. Enhanced levels of guanidinoacetate methyltransferase were found in the liver proteome (21). Guanidinoacetate methyltransferase, a major enzyme in creatine phosphate production, plays a protective role in the activities of Na+, K+-ATPase, and mitochondrial creatine kinase, as well as an antioxidant role in the prevention of lipid peroxidation and guanidinoacetate buildup (22). In addition, in camel brain cells, H^+^-ATPase is overexpressed. This helps camels to provide a rapidly usable source of energy supply and maintenance of cellular functions (21). The H+-ATPase (or V-ATPase) major purpose is to generate an electrochemical proton gradient across eukaryotic cell membranes, which energizes key cellular processes (57). Braking up and building up of H^+^-ATPase was described as the secret for life, owing to its importance in cell organelles functions (58).

## Enzymes Control the Camel Bull Behavior During the Rutting Season

Rutting camels showed elevated serum testosterone and 5α-dihydrotestosterone levels during breeding season as compared to other camels (23). There was elevated testosterone concentration in the plasma of testicular tissue in the breeding season (59–61). There was an increase in the volume of interstitial tissue (60) or an increase in Leydig cell number per testes (24) causing increased secretion of androgens during the breeding season. During the breeding season, there was an increase in the metabolism of androgen. Testosterone is converted to 5α-DHT through 5α-reductase pathway (62) or to estradiol (aromatizing pathway). In testis and poll glands higher activity of 5α-reductase was expressed and as a result, there is high production of 5α-DHT concentration in rutting camels. In eliciting a behavioral response, 5α-DHT is more reactive than testosterone (25) which suggests that in the expression of hormonal effects testosterone metabolism could be an essential step (63). Greater activity of 5α-reductase was presumably expressed in testes and poll glands, resulting in higher DHT concentrations in rutting camels (23). In rutting season, histological examination of the poll gland suggested that the gland is endocrine (64). The glands, during this time, displayed strong S100 protein, α-smooth muscle actin, and display immunoreactivity to keratin (65) which suggests secretory and contractile activity (66, 67). In rutting camels, the oxidative drug metabolism inhibition leads to elevating androgen production. In the same way, when testosterone is given to goats results in mixed-function oxidase inhibition (68). As compared to bulls, Cows are high metabolizers of Phase-1 metabolism (69). However, cytochrome P-450 was induced by androgenic hormones in rats (70). In the rutting period when camels are treated with 5α-DHT or testosterone, they depict no variation in the activity of phase-2 hepatic enzyme UDP-glucuonyl transferase. This means that only phase-I is affected by the androgens during its drug metabolism. Breeding season results in stimulation of poll gland with elevated 5α-DHT levels and α-reductase activity.

## Vimentin and Its Attractive Functions in Camels

Vimentin is an intermediate filament (IF) protein of type III that is expressed in mesenchymal cells. All mammal cells, as well as microorganisms, have IF proteins. The cytoskeleton is made up of intermediate filaments, tubulin-based microtubules, and actin-based microfilaments (71). Vimentin is important in maintaining and stabilizing the location of organelles in the cytoplasm. Vimentin is either laterally or terminally connected to the nucleus, endoplasmic reticulum, and mitochondria (72). Vimentin's dynamic nature is vital when it comes to providing flexibility to the cell. It is widely assumed that vimentin is the cytoskeletal component responsible for cell integrity. When cells were mechanically stressed, vimentin exhibited robustness not present in microtubule or actin filament networks (73).

**Figure 3:**
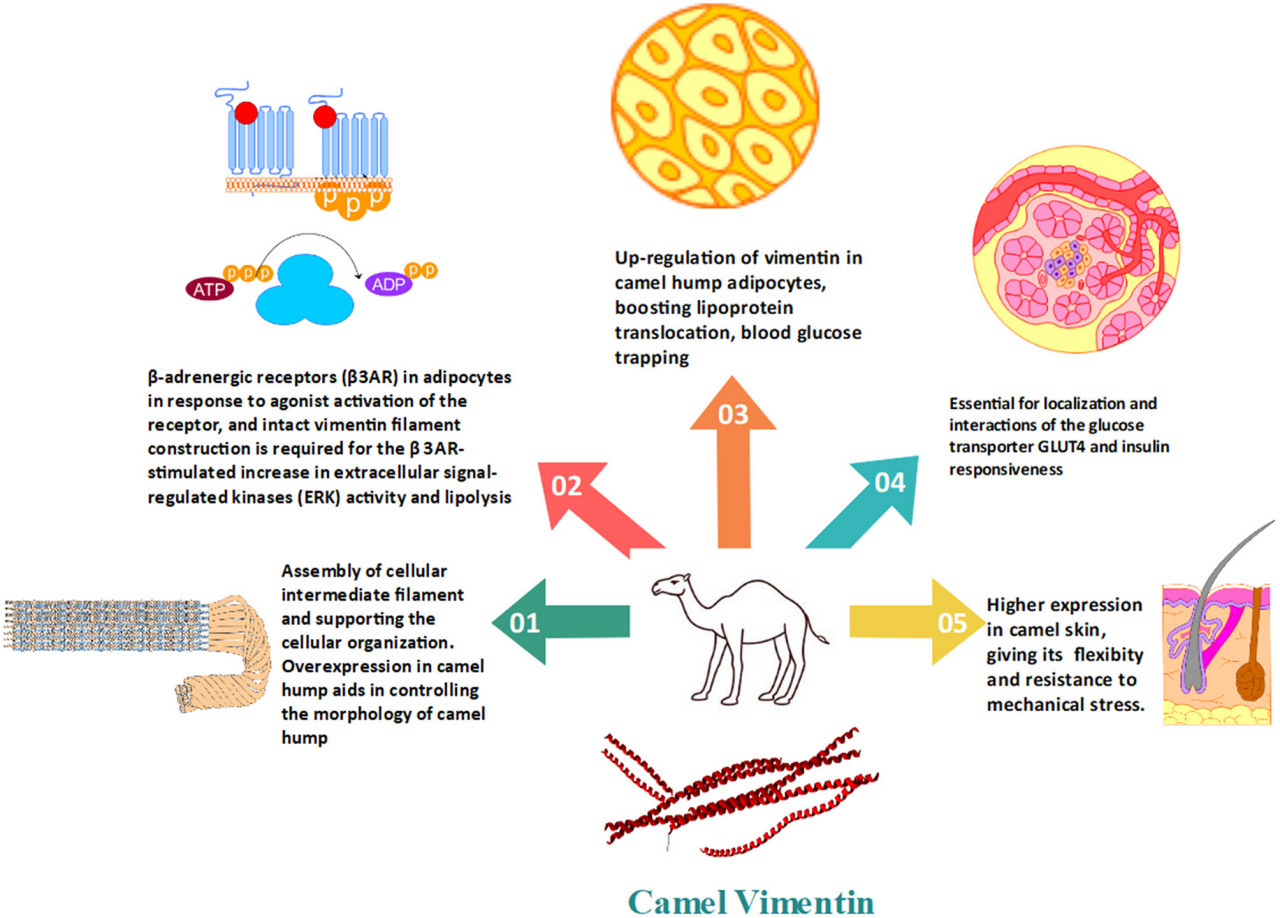
The importance of vimentin in the regulation of fat and glucose metabolism in camels as well as the conventional cytoskeleton formation.

Vimentin was found to be upregulated in the camel hump. Vimentin filaments create a cage-like structure surrounding the developing lipid droplet (Figure 3). The up-regulation of vimentin in adipocytes, boosting lipoprotein translocation, blood glucose trapping, and confronting external physical extra-stress, create a dynamic character for camel hump adipose tissue. The proper composition of the cytoskeleton including vimentin has been associated with the localization and interactions of the glucose transporter GLUT4 and insulin responsiveness (34). Vimentin in camels also helps in the process of inducing glucose adipocyte transport. This specific role of vimentin is performed in accordance with the elevated basal glucose levels (34, 74). These investigations have revealed the relevance and significance of adipocyte vimentin in humped camels' tolerance to high blood glucose levels. Vimentin is then thought to be an inducer of glucose trapping by adipocytes. This will help in camel adaptation to the harsh environment. Furthermore, camels have a high level of glucagon, which leads to an increase in baseline blood glucose, which is compatible with vimentin's participation in glucose transporter-induced glucose adipocyte transfer (34). These results point to adipocyte vimentin's modulatory role as a possible explanation for camels' high blood glucose tolerance. The upregulated vimentin in camel adipocytes is attributed to their adaptation in arid conditions to survive without water and food for a longer period, and it may aid the morphology of the well-nourished camel's hump (21). Vimentin connects with Catecholamine stimulation of β-adrenergic receptors (β3AR) in adipocytes in response to agonist activation of the receptor, and intact vimentin filament construction is required for the β 3AR-stimulated increase in extracellular signal-regulated kinases (ERK) activity and accelerating the lipolysis (26).

## Camel Tissues Have Multiple Forms of Drugs and Xenobiotics Metabolizing Enzymes

All animals are inevitably exposed to toxins as industrialization and living conditions change (or xenobiotics such as medications, insecticides, and industrial chemical pollutants). Water-soluble chemicals are expelled straight through the kidneys, however other chemical compounds are not, and lipophilic chemicals are biotransformed into more hydrophilic forms. Chemicals having higher hydrophilicity have a better excretion efficiency. Phase 1 and phase 2 enzymes are used to catalyze biotransformation processes. The essential enzymes in phase 1 reactions are CYPs and flavin-containing monooxygenase (FMOs). FMO enzymes, such as the CYP450 enzyme family, are thought to protect organisms from xenobiotics from the environment (27). Methimazole (MEM) and *N, N*'-dimethylaniline (DMA) metabolism by camel liver, kidney, brain, and intestine is dependent on FMO. In the microsomes of camel tissues, FMO-catalyzed metabolism was independent of cytochrome CYPs activity and showed the pH and temperature dependency typical of FMO enzymes. The oxidative metabolism of a wide range of xenobiotics, including nucleophilic nitrogen, sulfur, phosphorous, and selenium heteroatoms, is catalyzed by the microsomal nicotinamide adenine dinucleotide phosphate (NADPH) and molecular oxygen-dependent FMOs (75). In this context, camel FMOs were comparable to those of humans and rats that were completely independent of CYPs activities and imply the presence of multiple forms of drug-metabolizing enzymes in camel tissues.

## Enzyme Activity Under Various Mineral Environments

Trace elements have vital functions in the animal body since they are crucial components for numerous physiological processes that occur in them. Trace elements are responsible for the proper activities of various enzymes, the normal production of hormones, the synthesis of tissues, the equal transport of oxygen to all parts of the camel's body, the production of energy, the reproduction system of dromedaries and their growth. Despite the positive aspects, a lack of these trace elements could lead to the development of a variety of severe pathological disorders, including hormonal dysfunction, cardiac stress, various types of metabolic defects, and immunological abnormalities (76).

*Camelus dromedarius* lives largely in arid environments and prefers to forage on land. As a result, determining the availability of trace elements, plant content, and dry matter intake is very challenging. Several investigations have shown that, like other ruminants, these pseudo ruminants are susceptible to illnesses caused by a lack of trace elements at their typical levels (77). Many research papers have been published that identify clinical mineral deficits in dromedaries, however, these deficiencies are frequently overlooked because subclinical mineral deficiencies go unreported (78). Researchers investigated several physiological anomalies related to the metabolism of trace elements such as copper, zinc, iron, manganese, and selenium as a result of insufficient mineral feeding supplies and dromedary adaptation to severe and desert circumstances (79). Therefore, due to adaptations, their metabolic system usually adopts special mechanisms such as higher storage capacity of copper (28), during scarcity period more absorption of zinc and copper (29), maintenance of proper enzymatic activities in the under-nutrition period and tolerance for excess electrolytes and minerals like sodium, calcium and phosphorus was observed (30). Selenium (Se) deficiency was recorded and its associated disorders like cardiopathy were reported in camels. Young animals are more prone to Se deficiency which cause white muscle disease, discoloration of skeletal muscle and degenerative myocarditis in *Camelus dromedarius* (31).

The serum glutathione peroxidase activity in camels bears interesting features. The deficiency of Se leads to decreased glutathione peroxidase activity in both cows and camels. However, after supplementation of Se, the activity of the enzyme increased to reach a plateau in cows. In contrast, in camels, the activity of glutathione peroxidase continued to increase indefinitely, implying the ability of camel RBCs to store selenium during deficiency periods (31).

## Carbohydrase Enzymes in Camel

*Camelus dromedarius* is a typically abstemious mammal capable of surviving severe conditions such as the Sahara, where food is limited. As a result, specific characteristics of these animals have been found that facilitate their endurance. The dromedary includes a number of enzymes that are involved in the control and up-regulation of numerous biochemical processes.

The pancreas is the primary digestive organ of the body. It aids in the proper digestion of ruminal fermentation products, which also include some microbial cells and nutrients consumed. However, due to the considerable amounts of unfermented carbohydrates and protein exiting the rumen and moving toward the small intestine, extra consideration is required in this area following the advent of modern-day feeding strategies that stress the use of food concentrates (80). There is still a need to examine the pancreatic enzymes of camels in-depth, although relatively few research publications that describe the pancreatic enzymes have been published (32). Carbohydrate digestion in monogastric animals is generally accomplished by the action of hydrolytic enzymes that are generated in the animal's own body. However, in ruminants, carbohydrates are properly degraded by bacteria located in the rumen, and these animals also release certain polysaccharide digesting enzymes (81). The polysaccharide enzymes aim to finish the remaining phase of food absorption that was not completed by microbial activity (82). The *Camelus dromedarius* is usually contemplated as a pseudo ruminant (83), and different types of intestinal enzymes, including disaccharidases and polysaccharides like dextranase and amylase, have been reported in camels (32). In comparison to other animals such as sheep, buffalo, and cows, the pancreas of the camel has a high concentration of Carbohydrase enzymes such as sucrase, cellobiase, Glucoamylase, maltase, trehalase, and alpha-amylase. Starch is made up of two physically separate polysaccharides called amylose and amylopectin. Alpha-amylase is an endohydrolase found in adolescent salivary and pancreatic secretions that hydrolyzes internal α-1,4-glucoside linkages of amylose in the small intestine to produce solubilized linear maltose oligosaccharides and so aids in normal meal digestion (84). However, the complete absorption of maltose and oligosaccharides require glucose hydrolysis at the α-1,6 and α-1,4 glucosidic bonds of the non-reducing ends of starch oligomers (85). Food is finally broken down in camels by sucrase-isomaltase and maltase-glucoamylase, which are found in the mucosa of the dromedary's small intestine and are known as brush boarded anchored enzymes. Brush-border maltase glucoamylase's significant activity in the small intestine resulted in the breakdown of linear regions of starch contained in meals into glucose. Furthermore, the action of brush-border sucrase-isomaltase is similar to that of brush-border maltase glucoamylase in that both help in the digestion of starch bonds (86).

## Natural Insulin Resistance in Camels

**Figure 4:**
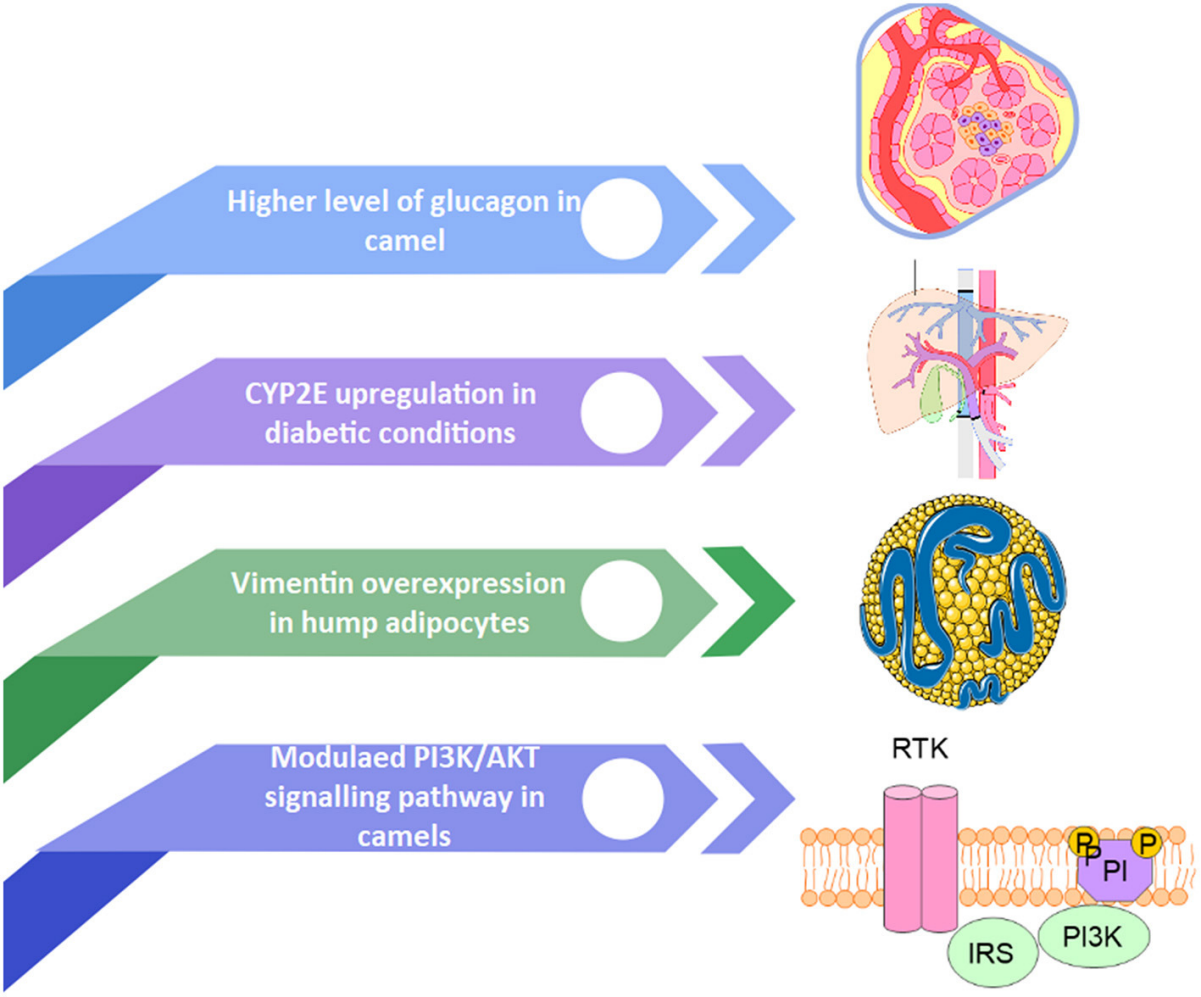
The potential mechanisms of insulin resistance in camels.

Ruminants, including camels, have higher blood sugar levels than non-ruminants (87). Previous research has found that elevated glucose levels in camels are caused by significant insulin resistance (88). Several mechanisms are suggested for such findings (Figure 4).

The CYPs 450 liver enzyme families are among the genes that evolve at a faster rate, it is reported that CYP2E is likely to be associated with type II diabetes Mellitus. In diabetic patients, the expression of CYP2E was upregulated in the liver (33). The greater CYP2E activity in camels is consistent with the concept of CYP2E overexpression in diabetes circumstances. Camels have a high glucagon level, which causes an increase in baseline blood glucose, which is consistent with vimentin's involvement in glucose transporter-induced glucose adipocyte transfer (34). In mice, a lack of vimentin avoids obesity and insulin resistance. Vimentin is involved in the insulin-dependent translocation of glucose transporter type 4 (GLUT4) to the plasma membrane, which is the most insulin-responsive glucose transporter isoform (89). The elevated expression of vimentin in camel hump adipocytes could contribute to peripheral insulin resistance.

In the insulin and type II diabetes signaling pathways in camels, there are about 21 upregulated genes (10). However, the precise mechanism of these genes' function in insulin resistance in camels is still being explored. PI3K and AKT are two of these genes that are elevated in camels. Meals trigger the PI3K/AKT signaling pathway, which enhances glucose consumption while decreasing gluconeogenesis in the liver and muscle, increases body lipid deposition, modulates lipid and glucose metabolism balance, and decreases appetite in the brain (90). Hence, it is suggested that elevated glucose levels in camel blood result from their high insulin resistance (88). When human and camel insulin receptors were compared, camel insulin receptors have a higher positive electrostatic potential in the insertion domains (ID), notably the ID-loop. The ID-α'~αCT'~ID-β is required for insulin receptor signal transduction (35). These changes in charges might affect the signal transduction efficiency of the insulin receptor in camels.

In the case of camels, there are contradictory circumstances between camel tissues, which have intrinsic insulin resistance, and camel milk, which includes a lot of insulin-like chemicals and reduces insulin resistance in diabetic camel milk consumers. More research is needed to understand the glucose metabolism and insulin sensitivity in these species.

## Conclusion

It is concluded that *Camelus dromedarius* or Arabian camel is multipurpose animal species utilized for food, protection, transportation, and by-products for many years. It is regarded as the most sustainable mammal on the earth that can withstand the extreme and harsh weather conditions of deserts. Various research studies on dromedary have reported that there are some unique and specific features in camels compared to other animals like goats, sheep and buffalo, which make them able to fight against scarcity and water loss conditions. Certain physiological adaptations have been found in camels that include more storage of fat content in their humps, more resistant erythrocytes than other animals, and their kidneys can conserve an extra amount of water. Apart from physiological adaptations, certain genetic adaptations have also been studied in camels, such as certain rapidly evolving genes that were further involved in the metabolism of different compounds, such as CYPs, which were involved in the metabolism of arachidonic acid, as well as different types of protein-coding genes that regulate the protein and lipid metabolisms. There has been the discovery of many genes in camels' bodies that have played a significant role to bring about traits that enable the camels to adapt for survival in the harsh and extreme conditions faced upon living in the desert. These modifications have been brought about regulating certain metabolic pathways. The discovery of the genes encoding the enzymes which regulate these pathways can help scientists to further know the basis and implications of these adaptations. Resolving the mystery of the mechanism of adaptation and the molecular processes associated with the camels' life is still a growing depository and full of secrets that need to be resolved by further investigations.

## Author Contributions

MK, AA-T, MMo, KV, and MMa designed research, performed research, collected data, analyzed data, and wrote the paper. SN performed research, collected data, and analyzed data. All authors revised the paper and approved submission.

## Funding

This work was supported by the Deanship of Scientific Research, Vice Presidency for Graduate Studies and Scientific Research, King Faisal University, Saudi Arabia [Project No. GRANT223].

## Conflict of Interest

The authors declare that the research was conducted in the absence of any commercial or financial relationships that could be construed as a potential conflict of interest.

## Publisher's Note

All claims expressed in this article are solely those of the authors and do not necessarily represent those of their affiliated organizations, or those of the publisher, the editors and the reviewers. Any product that may be evaluated in this article, or claim that may be made by its manufacturer, is not guaranteed or endorsed by the publisher.
