# Corrigendum: Camel proteins and enzymes: a growing resource for functional evolution and environmental adaptation

**DOI:** 10.3389/fvets.2024.1492459

**Published:** 2024-11-07

**Authors:** Mahmoud Kandeel, Abdulla Al-Taher, Katharigatta N. Venugopala, Mohamed Marzok, Mohamed Morsy, Sreeharsha Nagaraja

**Affiliations:** ^1^Department of Biomedical Sciences, College of Veterinary Medicine, King Faisal University, Al-Ahsa, Saudi Arabia; ^2^Department of Pharmacology, Faculty of Veterinary Medicine, Kafr El Sheikh University, Kafr El Sheikh, Egypt; ^3^Department of Pharmaceutical Sciences, College of Clinical Pharmacy, King Faisal University, Al-Ahsa, Saudi Arabia; ^4^Department of Biotechnology and Food Science, Faculty of Applied Sciences, Durban University of Technology, Durban, South Africa; ^5^Department of Clinical Sciences, College of Veterinary Medicine, King Faisal University, Al-Ahsa, Saudi Arabia; ^6^Department of Surgery, Faculty of Veterinary Medicine, Kafr El Sheikh University, Kafr El Sheikh, Egypt; ^7^Department of Pharmacology, Faculty of Medicine, Minia University, Minya, Egypt; ^8^Department of Pharmaceutics, Vidya Siri College of Pharmacy, Bengaluru, India

**Keywords:** camel, metabolism, adaptation, enzymes, proteins

In the published article, reference number 3 was incorrectly written as “Xiao X, Wu Z-C. Chou K-CJPo. A multi-label classifier for predicting the subcellular localization of gram-negative bacterial proteins with both single and multiple sites. *PLoS ONE*. (2011) 6:e20592. doi: 10.1371/journal.pone.00 20592.” It should be: “Ali A, Baby B, Vijayan R. From desert to medicine: a review of camel genomics and therapeutic products. *Front Genet*. (2019) 10:17. doi: 10.3389/fgene.2019.00017.”

In the published article, reference number 6 was incorrectly written as “Andersson L, Georges M. Domestic-animal genomics: deciphering the genetics of complex traits. *Nat Rev Genet*. (2004) 5:202–12. doi: 10.1038/nrg1294.” It should be “Macfarlane W, Morris R, Howard BJN. Turn-over and distribution of water in desert camels, sheep, cattle and kangaroos. *Nature*. (1963) 197:270–1. doi: 10.1038/197270a0 and Schmidt-Nielsen B, Schmidt-Nielsen K, Houpt Tt, Jarnum S. Water balance of the camel. *Am J Physiol*. (1956) 185:185–94.”

In the published article, reference number 11 was a duplicate of reference 10, and was incorrectly written as “Sequencing TBCG. communications ACJN. Genome sequences of wild and domestic bactrian camels. *Nat Commun*. (2012) 3:1202.” It should be renamed as reference 10, and read: “Bactrian Camels Genome Sequencing and Analysis Consortium: Jirimutu, Wang Z, Ding G, Chen G, Sun Y, Sun Z, et al. Genome sequences of wild and domestic bactrian camels. *Nat Commun*. (2012) 3:1202. doi: 10.1038/ncomms2192.”

In the published article, reference number 16 was incorrectly written as “De Genst E, Saerens D, Muyldermans S, Conrath K. Antibody repertoire development in camelids. *Dev Comp Immunol*. (2006) 30:187–98. doi: 10.1016/j.dci.2005.06.010.” It should be “Fesseha H, Desta W. Dromedary camel and its adaptation mechanisms to desert environment: a review. *Int J Zoology Stu*. (2020) 5:23–8.”

In the published article, reference number 19 was incorrectly written as “Rinaldi M, Ceciliani F, Lecchi C, Moroni P, Bannerman DDJVi. immunopathology. Differential effects of α1-acid glycoprotein on bovine neutrophil respiratory burst activity and IL-8 production. *Vet Immunol*. (2008) 126:199–210. doi: 10.1016/j.vetimm.2008.07.001.” It should be “Mohamed SA, Fahmy AS, Salah HA. Disaccharidase activities in camel small intestine: biochemical investigations of maltase-glucoamylase activity. *Comp Biochem Physiol B Biochem Mol Biol*. (2007) 146:124–30. doi: 10.1016/j.cbpb.2006.10.001.”

In the published article, reference number 20 was incorrectly written as “McKinley MJ, McBurnie MI, Mathai ML. Neural mechanisms subserving central angiotensinergic influences on plasma renin in sheep. *Hypertension*. (2001) 37:1375–81. doi: 10.1161/01.HYP.37.6.1375.” It should be “Macfarlane W, Morris R, Howard BJN. Turn-over and distribution of water in desert camels, sheep, cattle and kangaroos. *Nature*. (1963) 197:270–1. doi: 10.1038/197270a0 and Schmidt-Nielsen B, Schmidt-Nielsen K, Houpt Tt, Jarnum S. Water balance of the camel. *Am J Physiol*. (1956) 185:185–94.”

In the published article, reference 41, “Almathen F, Charruau P, Mohandesan E, Mwacharo JM., Orozco-terWengel P, Pitt D, et al. Ancient and modern DNA reveal dynamics of domestication and cross-continental dispersal of the dromedary. *Proc Natl Acad Sci U S A*. (2016) 113:6707–12. doi: 10.1073/pnas.1519508113” was incorrectly placed in the article. The correct sentence reads:

“The dromedary can live in dry regions, near mountains, and distant locations because of specific qualities and attributes that allow them to thrive in desert lands, mountains, and remote areas where food and water are scarce (41).”

In the published article, the following reference was omitted from **Camel's Unique Genomics**, paragraph 4: “Bahbahani H, Musa HH, Wragg D, Shuiep ES, Almathen F, Hanotte O. Genome diversity and signatures of selection for production and performance traits in dromedary camels. *Front Genet*. (2019) 10:893. doi: 10.3389/fgene.2019.00893.” The reference has now been added as follows:

“The complete genome sequence data of six Arabian Peninsula camels and the genotyping-by-sequencing data of 44 Sudanese camels (29 packing and 15 racing) were studied to assess their genome diversities, relationships, and possible signals of positive selection (11).”

The sentence “Different sorts of metabolic processes occur in camels' bodies, which are primarily governed by the correct activities …” should be followed by Mohamed et al. (19).

A correction has been made to **Introduction**, paragraph 1. This sentence previously stated:

“Camels, which are members of the Camelidae family, were originally discovered in northern America about 35 million years ago during the Eocene period (2).”

The corrected sentence appears below:

“Camels, which are members of the Camelidae family, were originally discovered in northern America about 35 million years ago during the Eocene period (2, 3).”

A correction has been made to **Phenotypical and Environmental Adaptation in Camels**, paragraph 3. This sentence previously stated:

“In hot weather, camels begin to sweat when the temperature reaches this level (25).”

The corrected sentence appears below:

“In hot weather, camels begin to sweat when the temperature reaches this level (17, 18).”

A correction has been made to **Carbohydrase Enzymes in Camel**, paragraph 2. This sentence previously stated:

“There is still a need to examine the pancreatic enzymes of camels in-depth, although relatively few research publications that describe the pancreatic enzymes have been published (85).”

The corrected sentence appears below:

“There is still a need to examine the pancreatic enzymes of camels in-depth, although relatively few research publications that describe the pancreatic enzymes have been published (32).”

A correction has been made to **Renal Blood Flow and Renal Vasodilatation**, paragraph 1. This sentence previously stated:

“Renal preglomerular vessels aid in water reabsorption and are necessary for life in desert environments. 19(S)-HETE is potent vasodilator of this vessel (56).”

The corrected sentence appears below:

“Renal preglomerular vessels aid in water reabsorption and are necessary for life in desert environments. 19(S)-HETE is potent vasodilator of this vessel (10).”

The authors apologize for this error and state that this does not change the scientific conclusions of the article in any way. The original article has been updated.

